# Model for End-Stage Liver Disease Excluding INR Is Associated with Poor Prognosis in Elderly Patients with Decompensated Heart Failure

**DOI:** 10.3390/biomedicines13082000

**Published:** 2025-08-18

**Authors:** Michał Jurkiewicz, Wioletta Szczurek-Wasilewicz, Michał Skrzypek, Jacek J. Jóźwiak, Mariusz Gąsior, Bożena Szyguła-Jurkiewicz

**Affiliations:** 1 Student’s Scientific Society, 3rd Department of Cardiology, Faculty of Medical Sciences in Zabrze, Medical University of Silesia, 40-055 Katowice, Poland; 2 2nd Department of Cardiology and Angiology, Silesian Center for Heart Diseases, 41-800 Zabrze, Poland; 3 Department of Pharmacology, Faculty of Medicine, University of Opole, 45-040 Opole, Poland; 4 Department of Biostatistics, Faculty of Public Health in Bytom, Medical University of Silesia in Katowice, 40-055 Katowice, Poland; 5 Department of Family Medicine and Public Health, Faculty of Medicine, University of Opole, 45-040 Opole, Poland; 6 3rd Department of Cardiology, Faculty of Medical Sciences in Zabrze, Medical University of Silesia, 40-055 Katowice, Poland

**Keywords:** Model for End-stage Liver Disease, heart failure, risk assessment, aged, prognosis, liver function tests

## Abstract

**Introduction:** Heart failure (HF) remains a leading cause of morbidity and hospitalization among elderly patients. Therefore, identifying reliable prognostic indicators is crucial for improving clinical outcomes in this population. The aim of this study was to evaluate the association between the Model for End-stage Liver Disease excluding INR (MELD-XI) and clinical outcomes in elderly patients hospitalized for decompensated HF. **Material and methods:** This was a single-center observational study involving 242 elderly patients with end-stage HF who were hospitalized for decompensation at our institution between 2019 and 2023. Upon hospital admission and discharge, MELD-XI scores were calculated using serum bilirubin and creatinine levels. The primary endpoint of the study was all-cause mortality during the follow-up period. **Results:** The median age of the patients was 68 years (66–74.6), and 78.9% were men. The median (Q1–Q3) follow-up time was 1.47 (0.78–2.31) years. During the follow-up period, 47.1% of the patients died. Independent predictors of mortality were diabetes mellitus [HR 1.656 (1.113–2.463), *p* = 0.013] and MELD-XI at discharge [OR 1.267 (1.210–1.327), *p* < 0.001]. The area under the receiver operating characteristic curves (AUC) for MELD-XI at discharge was 0.827 (95% CI: 0.776–0.878). The cut-off value for MELD-XI at discharge (>11.7 points) had a sensitivity of 97% and a specificity of 59%. **Conclusions:** Independent predictors of death in the analyzed population of elderly patients with decompensated HF were diabetes mellitus and MELD-XI at discharge.

## 1. Introduction

Heart failure (HF) in elderly patients is a complex syndrome that affects multiple organ systems. The prevalence of HF increases significantly with age, making it a leading cause of hospitalization and death among elderly patients [1,2]. The prognosis for elderly patients with HF is serious due to the presence of many coexisting conditions, such as hypertension, diabetes, coronary artery disease, anemia, and chronic kidney disease, which synergistically interact with the progression of HF and affect the outcomes [3]. Although many studies have identified key risk factors for HF in the general population, most large-scale clinical trials focus on middle-aged or younger individuals. Consequently, elderly patients, who constitute the majority of HF patients in real-world clinical settings, are often underrepresented in randomized controlled trials and overlooked in the development of evidence-based guidelines [4,5]. Furthermore, age-related physiological changes and multimorbidity may influence the clinical course of HF, and risk factors for worse outcomes in elderly patients may differ from those observed in younger patients [5,6]. Therefore, identifying reliable prognostic indicators is crucial for improving clinical outcomes in this population. From a pathophysiological point of view, cardiohepatic and cardiorenal interactions deserve special attention in the population of elderly patients with HF. In elderly patients with HF, chronic kidney disease (CKD) is prevalent and associated with increased morbidity and mortality [7,8,9]. Similarly, hepatic dysfunction in HF patients can manifest as congestive hepatopathy or acute cardiogenic liver injury, both of which are linked to adverse clinical outcomes [10,11]. An indicator that combines both hepato–cardiac and renal–cardiac interactions is the Model for End-stage Liver Disease excluding INR (MELD-XI score), which was originally developed to assess liver dysfunction in patients with end-stage liver disease [12]. MELD-XI is calculated based on simple and widely available laboratory parameters—serum creatinine and bilirubin concentrations and may be used in a wide population of patients with HF, also in those on anticoagulant therapy due to embolism or frequent coexistence of AF [11,13]. Previous studies have shown that MELD-XI can be a useful prognostic indicator in patients with advanced HF [14,15,16]; however, data on its clinical utility of MELD-XI in elderly patients with decompensated HF are poor. Therefore, the aim of this study was to assess the association between MELD-XI and clinical outcomes in elderly patients hospitalized with decompensated HF.

## 2. Material and Methods

**Figure 1:**
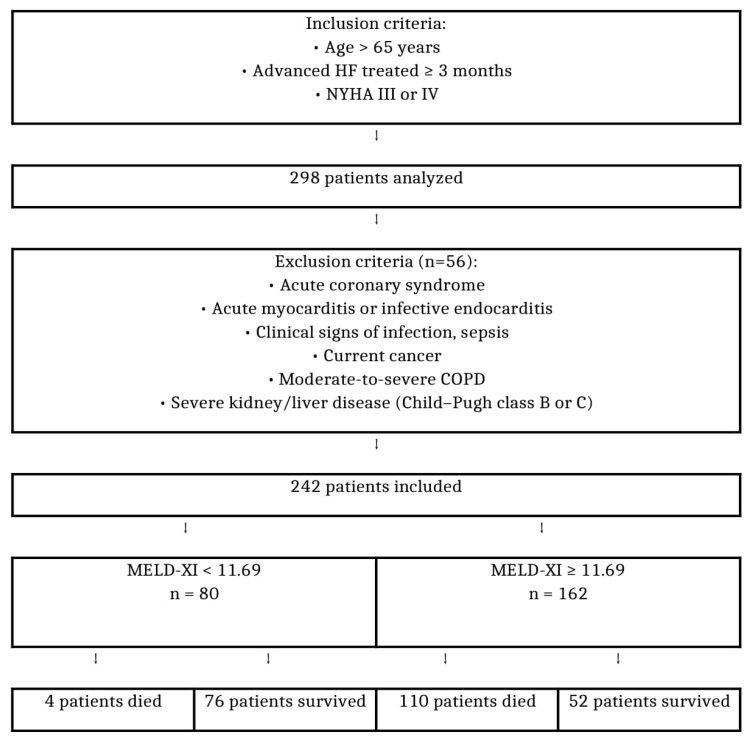
Flowchart of the study design for the inclusion and exclusion criteria. Abbreviations: HF, heart failure; COPD, chronic obstructive pulmonary disease; MELD-XI, Model for End-stage Liver Disease excluding INR, NYHA.

This single-center observational study enrolled consecutive elderly patients (aged >65 years) with advanced HF who were hospitalized for decompensation between 2019 and 2023 at our institution. HF was diagnosed by the attending physicians using a combination of clinical symptoms, physical findings, chest radiography, laboratory tests, and echocardiographic assessment. During the physical examination, we evaluated each patient’s resting pulse, weight, height, body mass index, presence of edema in the limbs, crepitus above the lungs, fluid in the pleural and the peritoneal cavity, and the presence of splenomegaly or hepatomegaly. HF exacerbation was defined according to the Framingham criteria as acute decompensation requiring urgent therapy and hospitalization [17]. The study design with the inclusion and exclusion criteria was presented in Figure 1.

At enrollment, all patients underwent a physical examination, anthropometric measurements, laboratory testing, and echocardiography.

For each patient, the MELD-XI score was calculated according to the following formula at the time of admission and discharge:

MELD-XI = 5.11 × ln (total bilirubin in mg/dL) + 11.76 × ln (creatinine in mg/dL) + 9.44

The minimum value for all biochemical parameters was standardized at 1.0 mg/dL, while the upper limit for serum creatinine concentration was capped at 4.0 mg/dL [12]. Creatinine and bilirubin concentrations required for calculating the MELD-XI scores were measured at the hospital’s central laboratory using the COBAS 6000 analyzer (Roche Instrument Center AG, Rotkreuz, Switzerland). The upper reference limit for total bilirubin was 21 μmoL/L, and for serum creatinine was 115 μmoL/L.

The analyzed cohort was divided into two groups based on the MELD-XI score cut-off point: a low MELD-XI score (<11.69) and a high MELD-XI score (≥11.69). Receiver operating characteristic (ROC) analysis was performed, and the optimal cut-off value for MELD-XI was determined using the Youden index.

The primary endpoint of the study was all-cause mortality during the follow-up period. Patients were followed up via telephone interviews and outpatient and readmission records were reviewed for potential cardiovascular events. Data on long-term outcomes, including the date of death, were obtained from the official registry of the National Health Fund. Follow-up was available for all patients. Written informed was obtained from all participants prior to their inclusion in the study. The research protocol received approval from the Ethics Committee of the Medical University of Silesia (approval codes: KNW/0022/KB1/53/18, dated 19 June 2018; and PCN/0022/KB1/20/I/21, dated 4 May 2021, PCN/CBN/0052/KB1/20/II/21/22 dated 20 September 2022).

### Statistical Analysis

Statistical analyses were conducted using SAS software version 9.4 (SAS Institute Inc., Cary, NC, USA) and R software version 4.4.3 (R Foundation for Statistical Computing, Vienna, Austria). Continuous variables were summarized as medians with corresponding interquartile ranges and assessed for group differences using the Wilcoxon rank-sum test. Categorical variables were expressed as counts and percentages and analyzed via the chi-squared test. Statistical significance of the difference between MELD-XI values on admission and discharge was assessed with the use of the Wilcoxon signed rank test. The optimal cut-off for the MELD-XI scores was determined based on the Youden index. Survival between analyzed groups (low-MELD-XI group vs. high-MELD-XI group) was compared using Kaplan–Meier curves and the log-rank test. The area under the receiver operating characteristic curve (AUC) was used to compare the ability of MELD-XI at both admission and hospital discharge to predict mortality over follow-up period. Diagnostic utility was evaluated using sensitivity, specificity, and accuracy. DeLong’s test was used to compare AUC values. A two-tailed *p*-value below 0.05 was considered indicative of statistical significance.

The Cox proportional hazards model was utilized to determine the hazard ratio (HR) and 95% confidence interval (CI) for death during the follow-up. Univariable Cox proportional hazards analysis was first performed to identify potential independent predictors of mortality for inclusion in the multivariable model. The relationships between explanatory variables were assessed using the Spearman rank correlation coefficient, and multicollinearity was evaluated using tolerance values and variance inflation factors. HRs were presented with 95% CIs, and a *p*-value < 0.05 was considered statistically significant.

## 3. Results

The baseline characteristics of the study population at admission are presented in Table 1. A total of 242 patients were analyzed, with a median age of 68 years (66–74.6), and 78.9% were men. NYHA functional class III was present in 54.1% of patients, while class IV was observed in 45.9%. The study population was categorized into two groups based on the MELD-XI cut-off at admission: Group A included patients with MELD-XI < 11.69, and Group B included those with MELD-XI ≥ 11.69. The median (Q1–Q3) follow-up time was 1.47 years (0.78–2.31), and the range was from 0.04 to 4.3 years. None of the patients were lost to follow-up. Two patients died during the index hospitalization. During the follow-up period, 4 patients (5.0%) in the MELD-XI < 11.69 group and 110 patients (67.9%) in the MELD-XI ≥11.69 group died (*p* < 0.001). The overall mortality rate during follow-up was 47.1%. In the high MELD-XI group (≥11.69), creatinine, bilirubin, and N-terminal pro-B-type natriuretic peptide (NT-proBNP) levels were significantly higher, while sodium levels were significantly lower compared to the low MELD-XI group (<11.69). Additionally, comorbidities such as hypertension and permanent atrial fibrillation were more prevalent in the high MELD-XI group (≥11.69) compared to the low MELD-XI group (<11.69). Regarding echocardiographic parameters, left atrial diameter and left ventricular end-diastolic dimension were significantly greater in patients with high MELD-XI scores (≥11.69).

Table 2 presents the key laboratory values at both admission and discharge in the analyzed population. During hospitalization, significant reductions were observed in serum bilirubin, creatinine, MELD-XI score, and N-terminal pro-B-type natriuretic peptide (NT-proBNP) levels, along with a modest improvement in estimated glomerular filtration rate (eGFR) (all *p* < 0.05).

**Figure 2:**
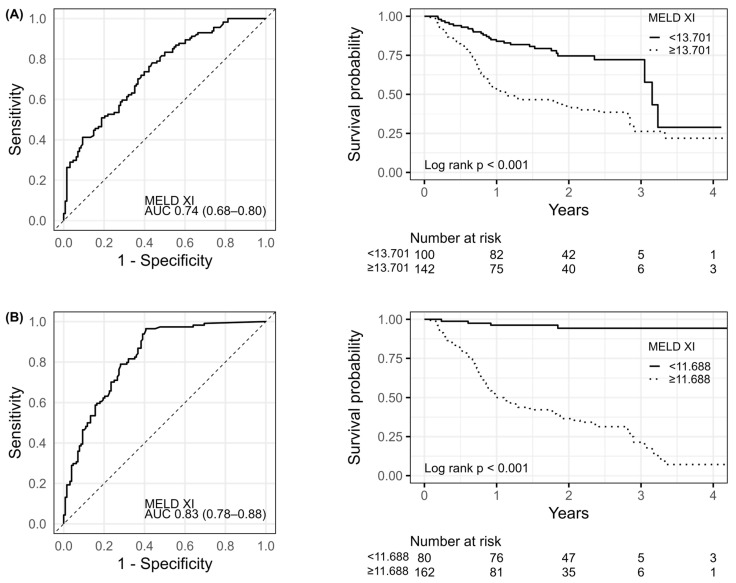
The ROC and Kaplan–Meier curves for MEDL-XI on admission (**A**) and discharge (**B**). Abbreviations: AUC, area under the curve, see Table 1.

The receiver operating characteristic curves (ROC) and Kaplan–Meier survival curves for MELD-XI on admission and discharge are presented in Figure 2. The summary of ROC curves analysis for MELD-XI on admission and at discharge is shown in Table 3. Patients with higher MELD-XI showed a worse prognosis than patients with lower MELD-XI [on admission 87 (61.3%) vs. 27 (27.0%); log rank *p* < 0.001; at discharge 110 (67.9%) vs. 4 (5.0%); log rank *p* < 0.001]. The AUC indicated acceptable discriminatory power of MELD-XI on admission and good prognostic power for MELD-XI at discharge for mortality. From the ROC curve analysis, a cut-off value of 13.70 for MELD-XI on admission yielded a sensitivity of 78% and specificity of 56%, while the cut-off value for of 11.69 for MELD-XI at discharge yielded sensitivity of 97% and specificity of 59%. The AUC for MELD-XI at discharge was significantly higher than that for MELD-XI at admission (AUC difference: 0.09 (95% CI: 0.03–0.14); *p* = 0.003).

In the multivariable analysis, independent predictors of death were diabetes mellitus [HR 1.656 (95% CI 1.113–2.463), *p* = 0.013] and MELD-XI score at discharge [OR 1.267 (95% CI 1.210–1.327), *p* < 0.001]. A summary of the univariable and multivariable analyses is presented in Table 4.

## 4. Discussion

Our study showed that independent predictors of death in the analyzed population are MELD-XI score at discharge and diabetes mellitus.

The most important finding of our study is that the MELD-XI score at discharge is independently associated with all-cause mortality, highlighting the negative influence of multi-organ dysfunction on clinical outcomes in elderly patients hospitalized with decompensated HF. Our study showed that patients with higher MELD-XI scores at discharge showed significantly higher all-cause mortality compared to those with lower MELD-XI scores. Furthermore, higher MELD-XI scores were associated with increased creatinine, total bilirubin, and NT-proBNP concentrations, as well as decreased sodium concentration, and a greater prevalence of comorbidities compared to the lower MELD-XI group.

Previous studies consistently showed that higher MELD-XI score is a marker of severe HF with organ dysfunction and is associated with worse outcomes [11,13,18,19]. Lin et al. found that MELD-XI score on admission independently predicted 3-year all-cause mortality in hospitalized elderly patients with HF [18]. However, this study differed from ours, as only 20% of patients had reduced left ventricular ejection fraction, whereas our analysis included only patients with end-stage HF and reduced LVEF. Similarly, in a multicenter study of aged patients with HF, Okano et al. found that those with elevated MELD-XI (≥11) had significantly more cardiovascular events, and MELD-XI remained an independent predictor of worse prognosis even after adjusting for conventional risk factors [11]. This study also analyzed patients with higher left ventricular ejection fraction (LVEF) (median 51.0) than in our study, and only 21% of patients were in NYHA class III/IV [11]. Kawahira et al. also showed that a higher MELD-XI score was independently associated with increased risk of all-cause mortality during a mean follow-up of 2.8 years in the elderly patients admitted for acute HF [19]. Furthermore, the combination of high MELD-XI and FIB-4 index improved further stratification of patients at higher risk of all-cause mortality, regardless of LVEF [19]. A recent study by Mizobuchi et al. investigated the prognostic value of the MELD-XI score at both admission and discharge in patients hospitalized for acute decompensated HF [20]. The authors found that elevated MELD-XI scores at admission, and persistent elevation at discharge, were significantly associated with poor outcomes, including higher rates of all-cause mortality and rehospitalization. In contrast, patients whose MELD-XI scores normalized during hospitalization had more favorable prognoses [20]. Our findings are consistent with those of Mizobuchi et al., but in our group, the median LVEF was 19.5 (15–23), whereas in Mizobuchi’s group, the median LVEF was 49 (34–62) [20]. The dynamic changes in MELD-XI score provides additional prognostic information, highlighting the importance of monitoring values of the MELD-XI score during the hospitalization to identify patients at higher risk. Biegus et al. also highlighted the prognostic value of the MELD-XI score both at admission and during the hospitalization. Moreover, an increase in the MELD-XI score during hospitalization was independently associated with higher 1-year mortality risk [21]. Another study also showed that in chronic HF patients receiving cardiac resynchronization therapy (mean age 67 ± 12), higher baseline MELD-XI (third tertile) was associated with older age, more comorbidities, and significantly reduced survival over 30 months [22].

From the pathological point of view, the assessment of cardiorenal and cardiohepatic interactions using the MELD-XI score may be a significant prognostic tool in elderly patients with decompensated HF [11,19]. This scoring system integrates serum creatinine and bilirubin levels, reflecting the interplay between renal and hepatic functions and its prognostic value, especially in patients undergoing anticoagulant therapy—common in the elderly HF population—exceeds that of the traditional MELD model, which includes the INR [8]. In acute heart failure, renal dysfunction results from a combination of elevated central venous pressure, which impairs renal perfusion, and reduces cardiac output, leading to decreased glomerular filtration rate [23,24]. Additionally, neurohormonal activation—particularly of the renin–angiotensin–aldosterone system (RAAS) and sympathetic nervous system—promotes vasoconstriction, sodium retention, and further renal dysfunction [25,26]. Furthermore, inflammatory cytokines and oxidative stress exacerbate tubular injury and contribute to progression of interstitial fibrosis leading to worsening kidney function [25]. Similarly, hepatic dysfunction and elevated bilirubin levels in acute HF arise from complex hemodynamic disturbances. HF leads to increased central venous pressure, which is transmitted backward into the hepatic veins, resulting in hepatic venous congestion [27]. Over the time, this can progress to centrilobular necrosis and fibrosis. Simultaneously, reduced cardiac output in HF diminishes hepatic arterial blood flow, leading to hepatic hypoperfusion, which exacerbates hepatocellular injury, particularly in the centrilobular regions, which are most susceptible to hypoxia [27,28,29]. The combination of hepatic congestion and hypoperfusion impairs bile formation and excretion, leading to cholestasis and elevated serum bilirubin [28,30]. Thus, cardiorenal and cardiohepatic interactions generate vicious cycles of injury, where HF affects kidney and liver dysfunction and vice versa [19,20,26,31]. The elderly population is particularly susceptible to those injuries due to age-related declines in organ reserve and the presence of comorbidities [11,32]. Thus, MELD-XI through the prism of liver and kidney dysfunction in geriatric population of HF can identify high-risk patients who might be underestimated by cardiac measures alone [11,21].

Another factor independently associated with mortality in our study group is diabetes mellitus. It is a common comorbidity in HF, with a prevalence ranging from roughly one-third to nearly one-half of cases [33,34]. Although diabetes mellitus has been consistently linked to increased cardiovascular morbidity and mortality in patients with chronic HF and reduced ejection fraction (HFrEF), its direct influence on prognosis following HF hospitalization remains uncertain [35,36,37,38]. Some analyses have shown that diabetes mellitus is significantly associated with poorer in-hospital and post-discharge outcomes among patients with acute HF [34,36,39,40]. In contrast, other investigations reported no significant relationship between diabetes mellitus and mortality in HF patients after adjusting for confounding factors [33,41]. Furthermore, data regarding the impact of diabetes mellitus on the prognosis in elderly patients with advanced HF are limited, as most studies include a broad age range without isolating older individuals. Formiga et al. investigated first-time acute HF admissions in patients ≥ 75 years and reported no significant difference in 1-year mortality between those with and without diabetes mellitus (both ~30% mortality rate) [33]. However, their cohort included patients across the HF spectrum regardless of ejection fraction (mainly with preserved LVEF) or comorbidity [33]. In contrast, the study by Cunha et al. found that diabetes mellitus was linked to an almost twofold higher 5-year mortality risk in chronic HF patients under 75 years, whereas no such association was observed in those aged 75 years or older [39]. Furthermore, Kong et al. also demonstrated that diabetes mellitus independently predicted higher all-cause mortality in HFrEF patients with a mean age exceeding 65 years [34].

From a pathophysiological point of view, diabetes mellitus and hyperglycemia can adversely affect the cardiovascular system and worsen HF, which is particularly significant in elderly patients with reduced physiological reserve. Chronic hyperglycemia has direct negative effects on the myocardium—promoting myocardial fibrosis, oxidative stress, and cardiomyocyte dysfunction [42,43]. Additionally, diabetes accelerates atherosclerosis and ischemic injury and induces other HF risk factors (such as hypertension, atherogenic dyslipidemia, thrombogenesis, and systemic inflammation), leading to progression of HF [35]. In acute decompensated HF, uncontrolled hyperglycemia was associated with worse outcomes, whereas achieving better glycemic control was beneficial [35,42]. Therefore, in elderly patients hospitalized with HFrEF, diabetes mellitus should not be regarded solely as a comorbidity but as a pathophysiologically active factor of disease progression and mortality. While relatively few studies have specifically analyzed HF populations aged ≥65 years, our findings highlight diabetes mellitus as an independent predictor of adverse prognosis in elderly patients with advanced HF. Optimizing diabetes and glycemic control in this high-risk group may represent an important therapeutic target to improve outcomes.

This study has several limitations. It was a single-center observational study, which may limit the external validity and generalizability of the findings. The relatively small sample size further reduces the statistical strength and reliability of the presented results. Although patients with a documented history of liver disease were excluded, subclinical or undiagnosed or subclinical hepatic dysfunction could not be definitely ruled out. Moreover, follow-up was performed without fixed time points, potentially affecting the consistency of data collection. Furthermore, the MELD-XI score was initially developed to evaluate patients with advanced hepatic dysfunction; however, its prognostic accuracy has not been comprehensively validated in cohorts with HF. Finally, the MELD-XI score does not incorporate several clinically confounding factors that may enhance risk stratification in this patient population. Prospective validation is needed to confirm the prognostic utility of the MELD-XI score in predicting all-cause mortality.

## 5. Conclusions

In conclusion, independent predictors of death in the analyzed cohort of elderly patients with decompensated HF were diabetes mellitus and the MELD-XI at discharge. The MELD-XI score at discharge serves as a valuable prognostic indicator in patients with decompensated HF, reflecting the extent of hepatorenal dysfunction. Its integration into clinical practice can enhance risk stratification, inform therapeutic strategies, and potentially improve patient outcomes. It seems that calculating the MELD-XI score at discharge may help identify elderly HF patients who need more aggressive management or monitoring.

## Figures and Tables

**Table 1:** Baseline characteristics of the study population.

Parameter	MEDL-XI < 11.69 n = 80 ^1^	MEDL-XI ≥ 11.69 n = 162 ^1^	*p* Value ^2^
Socioclinical Characteristics			
Age, years	67.0 (65.0–74.6)	68.5 (66.0–74.7)	0.104
Female, n (%)	17 (21.3%)	34 (21.0%)	0.962
Ischemic etiology of HF, n (%)	48 (60.0%)	92 (56.8%)	0.634
BMI, kg/m^2^	26.3 (22.8–29.9)	26.4 (24.2–30.2)	0.251
Hypertension, n (%)	30 (37.5%)	105 (64.8%)	<0.001
Type 2 diabetes, n (%)	22 (27.5%)	70 (43.2%)	0.018
Persistent AF, n (%)	37 (46.3%)	111 (68.5%)	<0.001
COPD, n (%)	4 (5.0%)	13 (8.0%)	0.386
Analytical Parameters			
Total bilirubin on admission, µmol/L	14.7 (10.2–22.4)	23.4 (17.3–31.2)	<0.001
Total bilirubin on discharge, µmol/L	11.4 (8.5–16.2)	19.5 (14.7–25.5)	<0.001
Creatinine on admission, µmol/L	96.0 (80.8–115.5)	132.0 (110.3–166.8)	<0.001
Creatinine at discharge, µmol/L	86.0 (76.8–97.3)	131.0 (113.0–153.0)	<0.001
MELD-XI on admission	11.1 (9.4–13.8)	16.1 (13.7–19.2)	<0.001
MELD-XI at discharge	9.5 (9.4–10.6)	15.5 (13.4–17.7)	<0.001
MELD-XI differences	1.2 (1.0–3.4)	0.3 (−1.8–2.9)	0.001
APTT, s	36.6 (31.6–41.5)	37.2 (32.5–44.4)	0.188
Uric acid, µmol/L	418.0 (316.0–559.3)	422.0 (340.0–527.0)	0.642
Cholesterol, mmol/L	3.6 (2.9–4.5)	3.5 (2.7–4.3)	0.181
LDL, mmol/L	2.2 (1.6–2.7)	1.9 (1.3–2.5)	0.131
Hemoglobin, mmol/L	9.2 (8.2–11.3)	9.9 (8.4–12.7)	0.171
WBC, ×10^9^/L	8.0 (6.2–9.0)	7.0 (5.6–9.0)	0.014
Platelets	203.0 (158.5–273.5)	181.0 (142.0–218.0)	0.002
NT-proBNP, pg/mL	4652.5 (1876.0–9421.0)	7562.0 (3545.0–12,962.3)	0.002
Sodium, mmol/L	138.0 (135.0–140.0)	136.0 (133.0–139.0)	0.017
LVEDd, mm	66.0 (61.0–73.0)	69.0 (65.0–76.0)	0.003
LA, mm	49.0 (46.0–54.0)	52.0 (48.0–57.0)	0.005
LVEF, %	20.0 (14.0–26.0)	18.5 (15.0–21.0)	0.129
Therapy			
ICD/CRT-D, n (%)	80 (100%)	162 (100%)	1.0
B-blockers, n (%)	74 (92.5%)	151 (93.2%)	0.839
Ivabradine, n (%)	16 (20%)	22 (13.6%)	0.194
MRA, n (%)	65 (81.3%)	134 (82.7%)	0.779
ACEI/ARB/ARNI, n (%)	63 (78.8%)	126 (77.8%)	0.863
Dapagliflosin/Empagliflosin, n (%)	56 (70.0%)	106 (65.4%)	0.477
Loop diuretics, n (%)	73 (91.3%)	157 (96.9%)	0.066
Inotropic at admission, n (%)	20 (25.0%)	50 (30.9%)	0.344
VKA, n (%)	20 (25.0%)	54 (33.3%)	0.186
Digoxin, n (%)	11 (13.8%)	55 (34.0%)	<0.001
Statin, n (%)	40 (50.0%)	89 (54.9%)	0.469
Acetylsalicylic acid, n (%)	23 (28.8%)	40 (24.7%)	0.498
NOAC, n (%)	31 (38.8%)	51 (31.5%)	0.261

^1^ Median (IQR) ^2^ Wilcoxon rank sum test. Abbreviations: ACEI, angiotensin-converting enzyme inhibitor, AF, atrial fibrillation; ARB, angiotensin receptor blockers, ARNI, angiotensin receptor/neprilysin inhibitor; BMI, body mass index; COPD; chronic obstructive pulmonary disease; CRT-D, cardiac resynchronization therapy with defibrillator; HF, heart failure; ICD, implantable cardioverter-defibrillator; LA, left atrium; LDL, low-density lipoprotein; LVEDd, left ventricular end-diastolic dimension; LVEF, left ventricular ejection fraction; MELD-XI, Model for End-stage Liver Disease excluding INR; MRA, mineralocorticoid receptor antagonist; NOAC, non-vitamin K antagonist oral anticoagulants; NT-proBNP, N-terminal pro-B-type natriuretic peptide; VKA, vitamin K antagonists; WBC, white blood cells.

**Table 2:** Selected laboratory parameters on admission and at discharge in the analyzed population.

Parameter	On Admission	At Discharge	*p*
Bilirubin	21.1 (14.6–29.8)	16.8 (11.9–23.0)	<0.001
Creatinine	121.5 (95.0–149.0)	113.0 (94.3–143.8)	0.011
MELD-XI	14.6 (11.7–17.9)	13.4 (10.7–16.5)	<0.001
NT-proBNP	6352.5 (2887.3–11,403.3)	4538.0 (2209.0–8991.0)	<0.001
Sodium	137.0 (134.0–140.0)	136.0 (134.0–138.0)	0.132
Hemoglobin	9.6 (8.33–12.5)	10.2 (9.7–10.8)	0.775

Abbreviations: see Table 1.

**Table 3:** The summary of ROC curves analysis for MELD-XI on admission and at discharge.

	AUC [±95 CI]	Cut-Off	Sens. [±95 CI]	Spec. [±95 CI]	Accuracy
MELD-XI on admission	0.742 (0.681–0.803)	13.70	0.781 (0.694–0.853)	0.562 (0.472–0.65)	0.665
MELD-XI at discharge	0.827 (0.776–0.878)	11.69	0.965 (0.913–0.99)	0.594 (0.503–0.68)	0.769

Abbreviations: AUC, area under the curve; CI, confidence interval, see Table 1.

**Table 4:** Univariable and multivariable analyses of factors associated with prognosis.

Parameter	Univariable Analysis		Multivariable Analysis	
	HR (95% CI)	*p*	HR (95% CI)	*p*
Arterial hypertension	1.975 [1.328–2.937]	<0.001		
Diabetes mellitus	1.517 [1.046–2.200]	0.028	1.656 [1.113–2.463]	0.013
Bilirubin on admission	1.035 [1.023–1.047]	<0.001		
Bilirubin at discharge	1.029 [1.021–1.038]	<0.001		
Creatinine on admission	1.005 [1.003–1.007]	<0.001		
Creatinine at discharge	1.014 [1.010–1.018]	<0.001		
MELD-XI on admission	1.114 [1.077–1.152]	<0.001		
MELD-XI at discharge	1.251 [1.193–1.312]	<0.001	1.267 [1.210–1.327]	<0.001
Platelets ↓ *	1.003 [1.000–1.006]	0.030		
Sodium ↓	1.062 [1.021–1.103]	0.002		
logNT-proBNP on admission	1.324 [1.075–1.629]	<0.008		
Left atrium	1.029 [1.005–1.053]	0.016		
VKA	1.468 [1.002–2.150]	0.049		
The lack of NOAC use	2.033 [1.302–3.165]	0.002		

Abbreviations: HR, hazard ratio; CI, confidence interval; see Table 1. * *p* < 0.05, ↓ per one unit decrease

## Data Availability

The data presented in this study are available on request from the corresponding author. The data are not publicly available due to privacy restrictions related to the rules in our institution.
